# Effect of Pharmacological Inhibition of the Catalytic Activity of Phosphatases of Regenerating Liver in Early T Cell Receptor Signaling Dynamics and IL-2 Production

**DOI:** 10.3390/ijms21072530

**Published:** 2020-04-05

**Authors:** Oscar Aguilar-Sopeña, Sara Hernández-Pérez, Sergio Alegre-Gómez, Patricia Castro-Sánchez, Alba Iglesias-Ceacero, John S. Lazo, Pedro Roda-Navarro

**Affiliations:** 1 Department of Immunology, School of Medicine, Universidad Complutense de Madrid, Spain and 12 de Octubre Health Research Institute (imas12), 28040 Madrid, Spain; oaguilar@ucm.es (O.A.-S.); sara.hernandezperez@utu.fi (S.H.-P.); sergioalegre@ucm.es (S.A.-G.); patricia.castro@ed.ac.uk (P.C.-S.); albaceacero@gmail.com (A.I.-C.); 2 Departments of Pharmacology and Chemistry, University of Virginia, Charlottesville, VA 22908, USA; lazo@virginia.edu

**Keywords:** immunological synapse, endosomal compartment, phosphatase of regenerating liver, TCR early signaling, cytokine production

## Abstract

We have previously shown the delivery of phosphatase of regenerating liver-1 (PRL-1) to the immunological synapse (IS) and proposed a regulatory role of the catalytic activity of PRLs (PRL-1, PRL-2 and PRL-3) in antigen-induced IL-2 production. Nonetheless, the expression in T cells and delivery to the IS of the highly homologous PRL-3, as well as the role of the catalytic activity of PRLs in antigen-induced early signaling, has not been investigated. Here, the expression of PRL-3 protein was detected in primary CD4 T cells and in the CD4 T cell line Jurkat (JK), in which an overexpressed GFP-PRL-3 fluorescent fusion protein trafficked through the endosomal recycling compartment and co-localized with PLCγ1 signaling sites at the IS. Pharmacological inhibition was used to compare the role of the catalytic activity of PRLs in antigen-induced early signaling and late IL-2 production. Although the phosphatase activity of PRLs was not critical for early signaling triggered by antigen, it seemed to regulate signaling dynamics and was necessary for proper IL-2 production. We propose that enzymatic activity of PRLs has a higher significance for cytokine production than for early signaling at the IS. However, further research will be necessary to deeply understand the regulatory role of PRLs during lymphocyte activation and effector function.

## 1. Introduction

Antigen-induced activation of CD4 T cells involves the formation of a specialized adhesion with antigen presenting cells (APCs), called the immunological synapse (IS). The mature IS comprises a central supramolecular activation cluster (cSMAC), which supports signal termination and cytokine secretion, a peripheral (p)SMAC, which contains integrins assuring proper cell-cell adhesion and a distal (d)SMAC, which contains actin cytoskeleton rearrangements required for sustained early signaling. In this way, the IS supports communication between lymphocytes and other hematopoietic cells to achieve full T cell activation and adequate effector functions [1].

Signaling by the T cell Receptor (TCR) and costimulatory molecules results in recruitment to the IS of different kinases and protein tyrosine phosphatases (PTPs), which are currently considered important not only to maintain the resting state of T cells but also to promote T cell signaling [2,3]. Phosphatases of regenerating liver (PRLs) PRL-1, PRL-2 and PRL-3, encoded by the genes *PTP4A1*, *PTP4A2* and *PTP4A3*, respectively, are dual-specific phosphatases, which promote proliferation, migration and invasion of cancer cells [4,5]. It has been proposed that there is a regulatory role of PRLs in the reorganization of the cytoskeleton [6,7,8,9], which is important during T cell activation and establishment of the IS and during effector functions of lymphocytes [10,11]. We have shown the polarization of PRL-1 to the IS and proposed a role of PRLs during T cell activation and effector function [12]. Supporting this hypothesis, here, we show the expression of PRL-3 in T cells and the distribution of an ectopically expressed GFP-PRL-3 fluorescent fusion protein to PLCγ1 signaling sites at the IS. In order to provide further insight into the regulatory role of PRLs during T cell responses, we compare early signaling stimulated by antigen and late IL-2 secretion in cells treated with a recently described potent inhibitor of the catalytic activity of these enzymes [13,14]. Our data suggest that the catalytic activity of PRLs regulates the dynamics of antigen-induced early signaling and has a critical role for an adequate IL-2 production.

## 2. Results and Discussion

### 2.1. Expression and Subcellular Distribution of PRL-3 in T Cells

**Figure 1:**
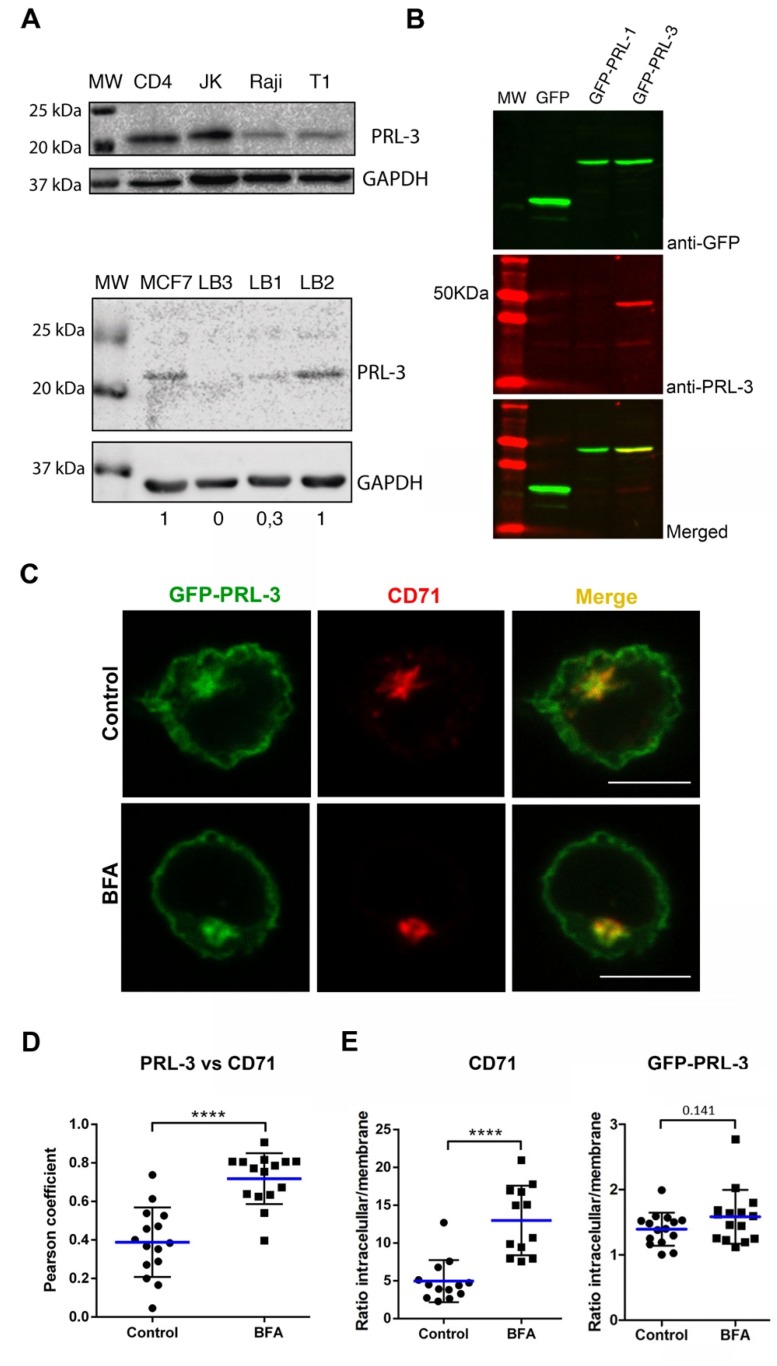
Expression and subcellular distribution of PRL-3. (**A**) Expression of endogenous PRL-3 assessed by Western blot. The cell type and antibody used are indicated. Lymphoblasts (LB) expanded from peripheral blood mononuclear cells (PBMCs) of three representative donors (LB1, LB2 and LB3) are shown. A lysate of MCF7 cell line was used as a positive control [20]. Numbers under the lower gels indicate the ratio PRL-3/GAPDH normalized to the positive control (**B**) Expression of the GFP-PRL-3, GFP-PRL-1 or GFP in JK cells analyzed by fluorescent Western blot. The green, red and merged images are shown. The antibody used in each channel is indicated. (**C**) Distribution of GFP-PRL-3 and CD71 in representative Jurkat (JK) cells treated or not (control) with 10 μg/mL Brefeldin A (BFA). The green and red channels, as well as merged images, are shown. Scale bar: 10 μm. (**D**) Pearson coefficients for assessing the co-localization of GFP-PRL-3 with CD71 in the endosomal compartment in JK cells treated or not with BFA. **(E)** Ratio of intracellular vs. plasma membrane CD71 or GFP-PRL-3. (**D** and **E**) Spots and squares indicate the individual cells analyzed. Blue lines indicate the average value. Control and BFA samples were analyzed by a two-tailed Student’s *t*-test. ****p < 0.0001.

The expression of PRL-3 has been very recently observed in peripheral blood mononuclear cells of both patients with acute lymphoblastic leukemia and healthy donors [15]. However, protein expression has not been evaluated in isolated primary CD4 T cells. PRL-3 expression was here detected by Western blot in Raji Burkitt’s lymphoma cells, Jurkat (JK) CD4 acute leukemia T cells, the T-B lymphoblast hybrid T1 cell line, resting peripheral blood CD4 T cells and in in-vitro Staphylococcus Enterotoxin E (SEE)-stimulated T cell lymphoblasts (Figure 1A). Together with our previous work showing the expression of PRL-1 and PRL-2 in human peripheral blood CD4 T cells [12], these data demonstrate the expression of PRLs in primary human lymphocytes and, consequently, suggest a regulatory role for this group of phosphatases in T cell biology. Interestingly, expression of PRL-3 was detected in the SEE-stimulated T lymphoblasts of six out of eight donors analyzed (Figure 1A), accordingly with different expression levels found in peripheral blood mononuclear cells (PBMCs) of different healthy donors by other authors [15]. Expression in Raji and JK cells is consistent with the described expression and role of PRL-3 in lymphoma and leukemia [15,16] and its role in these malignancies should be further investigated.

We have previously shown the traffic of PRL-1 to the IS in CD71-containing slow recycling endosomes, which polarize intracellular pools of the TCR to the IS [12,17]. Therefore, the traffic of the highly homologous PRL-3 was here investigated. We studied in JK cells the subcellular distribution of PRL-3 tagged with the green fluorescent protein (GFP-PRL-3). GFP-PRL-3 was expressed with the expected size and recognized by specific anti-PRL-3 immunoglobulins (Figure 1B). Consistently with data obtained for PRL-1, steady-state distribution of GFP-PRL-3 and CD71 revealed that GFP-PRL-3 trafficked to the recycling compartment (Figure 1C,D, control samples). We then addressed whether the recycling compartment had an active role in PRL-3 trafficking towards the plasma membrane. In order to study this issue, we took advantage of Brefeldin A (BFA), which inhibits the conventional secretory pathway [18] and blocks the surface expression of CD71 in T cells [19]. Consistent with the traffic of PRL-3 through the endosomal compartment, we observed higher co-localization between GFP-PRL-3 and CD71 after endosomal compartment compaction promoted by BFA treatment (Figure 1C,D). To evaluate the traffic of GFP-PRL-3 to the plasma membrane through the recycling compartment, we calculated in these samples the ratio of recycling compartment vs. plasma membrane protein. As expected, BFA clearly hampered the expression of CD71 at the plasma membrane, as revealed by the increment of this ratio. By contrast, BFA treatment had a weak effect on the plasma membrane localization of GFP-PRL-3 (Figure 1E). In concordance with this, immunofluorescence experiments showed that distribution of the endogenous PRL-3 to the membrane and the endosomal compartment was not affected by BFA treatment (Supplementary Figure S1). These data might indicate the existence of a transport of PRL-3 to the plasma membrane independent of the BFA-sensitive secretory pathway or a more stable half-life at the plasma membrane that should be investigated. Interestingly, the presence of PRL-3 in recycling endosomes suggests that PRL-3 molecules in transit through this endosomal compartment might be targeted to the IS during activation, as it has been previously shown for the TCR [17].

### 2.2. Delivery of GFP-PRL-3 to the IS

**Figure 2:**
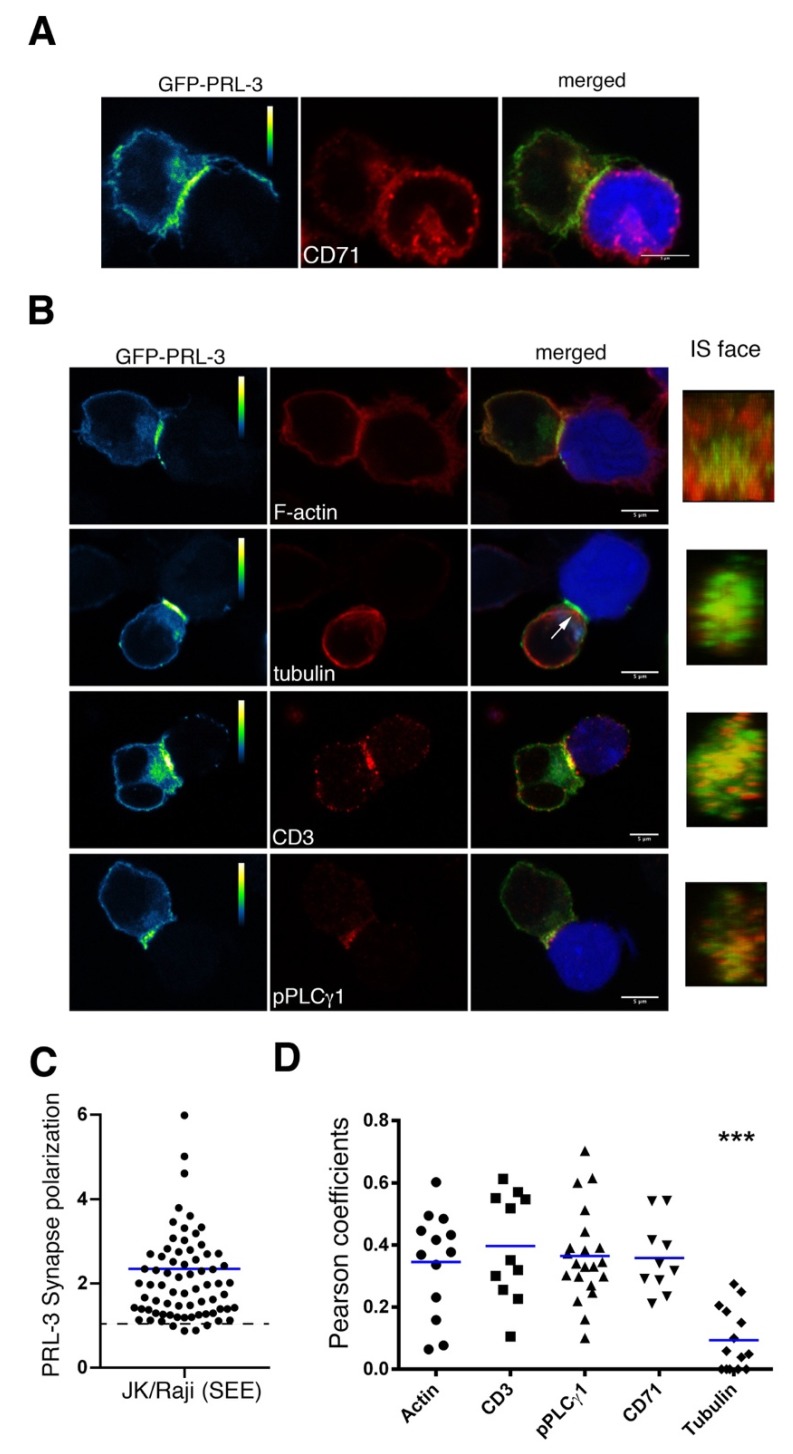
Distribution of GFP-PRL-3 to the immunological synapse. (**A**,**B**) Representative cell conjugates of JK cells interacting with SEE-loaded and CMAC (blue) labelled Raji cells. The green (pseudocolor) and red channels as well as the merged images are shown. The molecule stained and observed in the red channel is indicated. Calibration bar of the pseudocolor is indicated. Scale bar: 5 μm. The IS face obtained from the 3D reconstructions is shown. A white arrow points to the MTOC polarized to the IS. (**C**) Synapse polarization of GFP-PRL-3 assessed from the increment in fluorescence at the IS in comparison with the rest of the cell. Dots indicate individual cells analyzed and the blue line the average value. The dashed line indicates the ratio equals 1, meaning no polarization. (**D**) Pearson coefficients for assessing the colocalization of GFP-PRL-3 with the indicated molecule at the IS. Symbols indicate the analyzed cells and blue lines average values. Samples were compared by an ordinary one-way ANOVA with a Tukey’s multiple comparison test. Samples stained for tubulin were statistically different. ***p < 0.001.

The distribution of GFP-PRL-3 to the IS was studied in cognate interactions established by JK cells transfected with GFP-PRL-3 and SEE-loaded Raji APCs. To investigate the localization of GFP-PRL-3 in the polarized recycling compartment at the IS, JK and Raji cells were allowed to interact during 20 min in order to established mature interactions, which were then stained for CD71. Confocal microscopy showed a clear accumulation of GFP-PRL-3 at the IS (Figure 2A–C), where it co-localized with the polarized recycling compartment (Figure 2A,D). In concordance, time-lapse confocal microscopy showed the co-localization of GFP-PRL-3 and mCherry-CD3ζ at the endosomal compartment polarized to the IS (Supplementary Figure S2 and movie 1). Accumulation of GFP-PRL-3 was not observed in specimens containing JK cells interacting with Raji cells non-loaded with SEE, indicating that the observed accumulation was specific to SEE cognate interactions (Supplementary Figure S3). The traffic of PRL-1 and PRL-3 to the endosomal compartment and the IS suggests that these enzymes might regulate the secretion of cytokines, in particular those secreted to the IS, such as interleukin-2 (IL-2) [21]. Nevertheless, PRLs might also regulate the delivery to the IS of intracellular pools of the TCR or signaling molecules Lck and LAT, which also travel to the IS in the endosomal compartment [17,22].

The distribution of the accumulated GFP-PRL-3 at the mature IS was further analyzed by staining α-tubulin, which allows tracking the polarization of the microtubule organizing center (MTOC), and F-actin and CD3ε, which allow seeing the organization of the dSMAC and the cSMAC, respectively. To address the localization of GFP-PRL-3 regarding early T cell activating signaling, cell conjugates were also stained for active PLCγ1. 3D confocal microscopy implemented to visualize the face of the IS showed that GFP-PRL-3 distributed inside the dSMAC, where it partially co-localized with CD3ε, F-actin and with sites of active PLCγ1 signaling, as assessed by Pearson‘s coefficients. By contrast, a lower co-localization was found in samples stained for α-tubulin (Figure 2B,D).

Together, GFP-PRL-3 seems to traffic to areas of the IS above the tubulin cytoskeleton, including traffic in the endosomal compartment towards the cSMAC and traffic to peripheral areas, where perhaps it might have a role in pPLCγ1 signaling or in other processes such as the contraction of actomyosin arcs or adhesion. Microscopy methods with higher spatial and temporal resolution will enable us to further track the spatial and temporal regulation of PRL-3 at the IS. Additionally, perturbing its function will assist in our understanding of the role of PRL-3 in molecular dynamics at the IS.

### 2.3. Catalytic Activity of PRLs Regulates the Dynamics of Antigen-Induced Early Signaling and IL-2 Secretion

**Figure 3:**
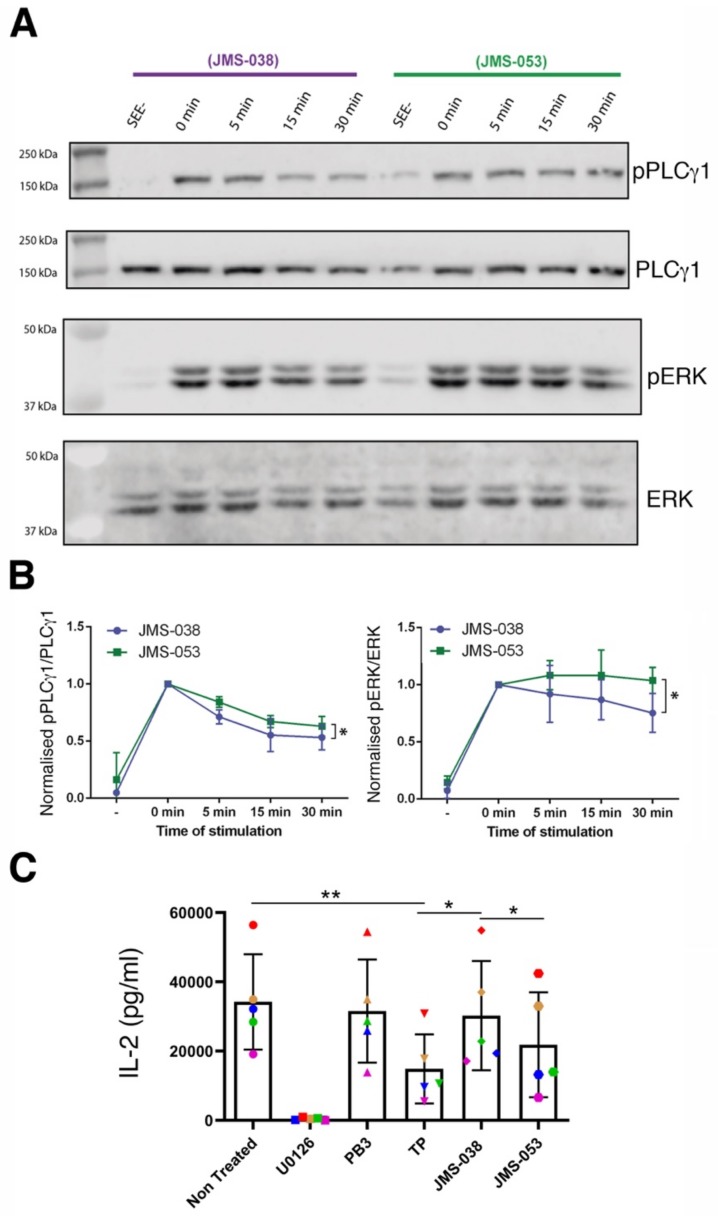
Regulatory role of the catalytic activity of PRLs during T cell activation. (**A**) Western blot of protein extracts of JK cells stimulated with SEE-loaded Raji cells for the indicated times in minutes (min). Samples of 0 min correspond to stimulation times at around the minute required to mix and spin-down cells before protein extraction. SEE- indicates the incubation of JK cells with Raji cells without SEE for the longest time used in stimulated samples. Molecules analyzed are indicated. (**B**) Phosphorylated fraction of the analyzed molecules in (A) normalized to the maximum. The mean +/− the standard deviation is shown (*n* = 3 independent experiments). Samples were compared by a paired two-tailed Student’s t-test. *p < 0.05. (**C**) IL-2 production by JK cells treated with 20 μM U0126, 25 μM PB3, 20 μM TP, 5 μM JMS-038 or JMS-053 and stimulated over night with PMA/Io. The mean +/− the standard deviation is shown (*n* = 5 independent experiments). The results of individual experiments are also indicated color coded in the graph. Samples were compared by a one-way ANOVA with a Tukey’s multiple comparison test. *p < 0.05, **p < 0.01.

The role of the catalytic activity of PRLs in early signaling and late cytokine secretion were compared in JK cells, which express all PRLs [12] (Figure 1A). PLCγ1 activation and IL-2 secretion were studied in cells treated with JMS-053, an inhibitor of the catalytic activity of PRLs, and JMS-038, the inactive compound serving as a negative control [13,14,23]. JK cells were activated at 37 °C with SEE-loaded Raji cells for 0, 5, 15 and 30 min and cell extracts analyzed by Western blot. The fraction of active signaling molecule revealed that the activation of both PLCγ1 and the downstream signaling molecule ERK were maximal at the earliest times in control samples (Figure 3A). Although no clear differences in the amplitude of the response were found between treatments, normalization to the earliest stimulation time showed a slightly more sustained activation of PLCγ1 and ERK1/2 in cells treated with the JMS-053 inhibitor (Figure 3A,B). Consistent results were obtained when experiments were done with primary T cell lymphoblasts stimulated with SEE-loaded Raji cells (Supplementary Figure S4). Thus, these data indicate that while the catalytic activity of PRLs is not critical for the early signaling induced by antigen in T cells, it seems to control in some extent the dynamics of this process. By contrast, treatment of JK cells with JMS-053 decreased the production of IL-2 induced by PMA and ionomycin stimulation, which bypasses early TCR signaling (Figure 3C). As expected, the MEK inhibitor U0126 indicated the dependence of IL-2 production in MAPK signaling. Consistent with our previous data obtained in peripheral blood T cells [12] and in the treatment with JMS-053, thienopyridone (TP), which also inhibits the catalytic activity of all PRLs [24], produced a more drastic reduction of the IL-2 production than procyanidin B3 (PB3), a more selective inhibitor of PRL-1 [25] (Figure 3C). TP, JMS-038 and JMS-053 compounds are structurally related [23] and consequently the effect of TP was also higher than the effect of the control compound JMS-038.

The previously reported regulatory role of PRL-1 and PRL-3 in the cytoskeleton [6,7,8,9] might explain the effect of the JMS-053 inhibitor on antigen-induced early signaling dynamics and on IL-2 levels in cell supernatants, perhaps the latter as a consequence of reduced secretion of cytokine-containing vesicles. The weak impact of the inhibition of the catalytic activity in early signaling might indicate an indirect effect. In this regard, PRL-3 dephosphorylates PIP_2_ [8] and this might constitute a regulatory step during early signaling. We cannot rule out the possibility that altered early signaling dynamics might be due to indirect effects of compounds in Raji cells. By contrast, when the IL-2 secretion was studied, we assured the effect of inhibitors on JK cells by stimulating with PMA and ionomycin. The lower inhibitory effect on IL-2 secretion of selective inhibition of PRL-1 than generic inhibition of PRLs in primary peripheral blood CD4 T cells [12] or JK cells (Figure 3C), points to a redundant role of these enzymes. Supporting this idea, a redundant role of PRL-1 and PRL-2 in spermatogenesis has been described in the mouse model [26]. It is also important to note that these enzymes might carry out functions non-dependent on the catalytic activity [27] that are expected to remain working under treatments used in this report. PRLs have been proposed to interact with cyclin M (CNNM) magnesium regulators [5], and due to the significance of this cation to T cell activation [28], it is tempting to speculate a role of such an interaction during IS assembly. The generation of specific inhibitors for each PRL, the use of specific functional genomic approaches and the identification of specific substrates in human T cells will help us to investigate whether or not these enzymes have a redundant role and phosphatase-independent functions during T cell immune responses. In any event, the catalytic activity of PRLs seems to regulate early signaling dynamics and IL-2 production in JK cells. It is expected that the identification of the molecular mechanisms mediating the regulatory role of PRLs on the cytoskeleton will enable us to understand the role of PRLs in lymphocyte activation and effector function. We predict that the enzymatic activity of PRLs will modulate adaptive immune responses.

## 3. Materials and Methods

### 3.1. Cells

Peripheral blood mononuclear cells (PBMCs) were obtained by blood centrifugation on LymphoprepTM solution. Blood was obtained from buffy coats processed at the transfusion center of the ‘Comunidad de Madrid’, Spain. For lymphoblast expansion, PBMCs were cultured in RPMI 1640 supplemented with 10% FCS, 2 mM L-Glutamine, 100 U/mL penicillin and 100 μg/mL streptomycin in the presence of SEE 1 µg/mL and IL-2 (50 U/mL). CD4 T cells were isolated from PBMCs by negative selection with DynabeadsTM UntouchedTM Human CD4 T cells kit (Invitrogen CA, USA). The CD4 T cell line Jurkat was cultured in RPMI 1640 medium supplemented with 10% FCS, 2 mM L-Glutamine, 100 U/mL penicillin, 100 μg/mL streptomycin, 1 mM sodium pyruvate and non-essential amino acids. The B cell line Raji was used as an antigen-presenting cell and the T/B lymphoblast hybrid T1 [29] was cultured in RPMI 1640 supplemented with 10% FCS, 2 mM L-Glutamine, 100 U/mL penicillin and 100 μg/mL streptomycin. Quality of cell lines and lymphoblast expansion, as well as purification efficiency of CD4 T cells and expression levels of CD71, were checked by flow cytometry with a FACSCalibur system (Becton Dickinson, Franklin Lakes, NJ, USA). FACS data was analyzed by the Flowjo software (Becton Dickinson, Ashland, OR, USA). Primary cell isolation in the project was approved by the Clinical Research Ethical Committee of the San Carlos Clinical Hospital in Madrid (Spain) (Favorable report number C.P.-C.I. 16/510-E_BS, 2016 November the 10th).

### 3.2. Antibodies and Reagents

APC-labeled anti-CD3 was obtained from BD Pharmingen (USA) and FITC-labelled anti-CD4 and the APC and FITC isotype controls were from Immunostep S.L (Spain). Rabbit anti-phosphoY783-PLCγ1, anti-PLCγ1 and anti-phosphoT202/T204-ERK1/2, as well as mouse anti-α tubulin were from Cell Signaling Technologies (Danvers, MA, USA). Mouse anti-ERK and anti-CD71 were from BD Biosciences (USA). Mouse anti-GAPDH was from Bio-Rad ( Hercules, California, USA), mouse anti-PRL3 from Santa Cruz Biotechnology (USA) and mouse anti-CD3ε (T3b clone) was provided by Dr. Francisco Sanchez-Madrid (Hospital Universitario de la Princesa, Madrid, Spain). A total of 680 goat-anti rabbit and 800 goat-anti mouse IR dyes were from Miltenyi Biotec (USA). Anti-rabbit and anti-mouse horseradish peroxidases were from Milipore (Burlington, Massachusetts, USA) and goat anti-mouse-Ig Alexa594 and goat anti-rabbit-Ig Alexa594 were from Molecular Probes (USA). Poly-L-Lysine was obtained from Sigma Aldrich ( St. Louis, Missouri, USA), and Phalloidin-594 and the fluorescent tracker chloromethyl derivative of aminocoumarin (CMAC) were obtained from Molecular Probes. Thienopyridone (TP) was obtained from Enamine (Kyiv, Ukraine), procyanidin B3 (PB3) from Chem Faces (Wuhan, China) and the MEK inhibitor U-0126 from Cell Signaling. JMS-053 and JMS-038 molecules were provided by John Lazo (University of Virginia, Charlottesville, VA, USA). Brefeldin A was obtained from Sigma Aldrich, lymphoprepTM from Rafer (Spain), laemli buffer from Alfa Aesar (Ward Hill, Massachusetts, USA), Staphylococcus Enterotoxin E (SEE) from Toxin Technologies (Saratosa, FL, USA) and PMA and ionomycin from Sigma Aldrich.

### 3.3. Plasmids and Transfection

GFP-PRL-1 expression vector was previously described [12]. The mCherry-CD3ζ plasmid was kindly provided by Dr. Alarcon (Molecular Biology Centre, CSIC, Madrid, Spain). PRL-3 cDNA was amplified by RT-PCR and cloned at the XhoI and BamHI restriction sites of the peGFP-C1 vector from Clontech Laboratories (USA). The resulting construct was sequenced and the expression of the GFP-PRL-3 fluorescent fusion protein verified by Western blot of transfected JK cells (Figure 1B). JK cells were nucleofected with the plasmid GFP-PRL-3 or a mixture of GFP-PRL-3 and mCherry-CD3ζ using the Amaxa NucleofectorTM II system and the AmaxaTM Cell Line Nucleofector Kit V (Lonza, Switzerland). Alive cells were obtained by centrifugation in lymphoprepTM solution.

### 3.4. Stimulation of JK Cells for Western Blot and ELISA Assays

For intracellular signaling studies by Western bot, JK cells were incubated at 37 °C with SEE-loaded Raji cells at a JK/Raji ratio 10:1 for the times indicated in experiments. The stimulation at time 0 min corresponds to JK cells mixed with SEE-loaded Raji cells, and immediately centrifuged and lysed (this resulted in a time of around one minute at room temperature, which is enough stimulation time for activating early signals to proceed). For the analysis of the IL-2 production, JK cells were stimulated overnight with 10 ng/mL PMA and 1 μM Ionomycin (PMA/Io) before collecting the supernatants for ELISA assays. When specified, cells were pre-incubated for 1 h with the indicated inhibitor, which was maintained during the whole stimulation time.

### 3.5. Western Blot

Cells were lysed for 30 min in ice-cold lysis buffer containing 20 mM Tris-HCl pH 7,5; 1% NP-40; 0,2% Triton X-100; 2 mM EDTA; 150 mM NaCl; 1,5 mM MgCl2; 5 mM β-glicerolphosphate; 1x protease inhibitor cocktail; 1 mM NaF; 1 mM PMSF; 1 mM Na3VO4 and 1 mM Sodium pyrophosphate. Lysate were then centrifuged at 10,000 rpm for 10 min at 4 °C and soluble fractions were collected, mixed with 6× Laemmli buffer containing 20% of β-mercaptoethanol, boiled at 95 °C for 5 min and resolved in 10% SDS-PAGE acrylamide gels. Resolved proteins were transferred to Immobilion-FL membranes. Membranes were blocked with LICOR blocking buffer or TBS-BSA 5%, incubated overnight with primary mouse or rabbit antibodies and incubated 30 min with anti-mouse or rabbit secondary antibodies. All blots were scanned and fluorescence (pERK/ERK) or chemiluminiscence (pPLCγ1/PLCγ1) was quantified with an Odyssey Infrared Imager (LICOR, USA). For chemiluminiscence, western Blot was revealed with PierceTM ECL Plus Substrate (Thermo Fisher Scientific, USA) using Odyssey Infrared Imager. Densitometry of images was done with Image Studio Freeware (LICOR). Blots were striped in 50 mL containing 2% SDS; 12,5% Tris-HCl pH 6.8 and 0.7% β-mercaptoethanol for 30 min at 50 °C.

### 3.6. Enzyme-Linked Immunoabsorbent Assay (ELISA)

The IL-2 content in supernatants was determined by ELISA assays using the BD OptEIA ELISA set (Becton Dickinson). Quantification was done in an ELx800 absorbance microplate reader (Biotek, USA). The non-treated cells contained the vehicle (DMSO) for TP, U0126 and PB3 treatments. The compound JMS-038 was used as control for the JMS-053 inhibitor [13].

### 3.7. Immunofluorescence and Confocal Microscopy

JK cells were conjugated at a cell ratio 1:1 with Raji cells loaded with 1 μg/mL SEE (or unloaded in control samples) and labeled with 10 μM CMAC for proper identification. Conjugates were allowed to interact for around 20 min on poly-l-Lysine-coated coverslips. These cell conjugates or JK cells alone were fixed and permeabilized as previously described [12]. Samples were then stained with the indicated primary antibodies at RT for 1 h followed by incubation with corresponding secondary Alexa594-conjugated antibodies at RT for 30 min. Samples were mounted in mowiol before microscopy. In BFA assays, JK or T1 cells were treated during 4 h with BFA before fixation/permeabilization and staining.

Confocal microscopy of fixed samples and in-vivo conjugates in time-lapse experiments was performed with a FV-1200 microscope (Olympus Deutschland GmbH, Germany). Excitation lines of length 405 nm (for the CMAC), 488 nm (for the GFP) and 594 nm (for the Alexa594 and mCherry) were used. An elapsed time of 10 s was used in time lapse microscopy. For 3D reconstructions z-stacks were acquired every 0.3 μm. Pearson coefficients for assessing co-localization, accumulation of GFP-PRL-3 at the IS, the 3D reconstruction and the ratio of intracellular vs. plasma membrane protein was performed with ImageJ freeware (National Institutes of Health). The data for T1 samples and control JK/Raji (SEE-) were acquired with a Zeiss Axio Imager. A1 fluorescence microscope coupled to a LED source of light LED:pE-300 (Cool LED) by using and objective EC Plan-Neofluar 63x/1,25 Oil M27.

### 3.8. Statistical Analysis

Statistical analysis was implemented in PRISM 6 (GraphPad Software, San Diego, California, USA). The used tests and the corresponding *p*-values are indicated in figure legends.

## Supplementary Materials

Supplementary materials can be found at https://www.mdpi.com/1422-0067/21/7/2530/s1.
