# Supplementary material for: Effect of Pharmacological Inhibition of the Catalytic Activity of Phosphatases of Regenerating Liver in Early T Cell Receptor Signaling Dynamics and IL-2 Production
Source: Int J Mol Sci. 2020 Apr 5;21(7):2530. doi: 10.3390/ijms21072530 (PMC7177812; doi:10.3390/ijms21072530)
Supplement: Supplementary file 1 [file ijms-21-02530-s001.zip › New_revised_figures/Supplementary_figures/Supplementary figure and movie legends.docx]

**Supplementary Figure 1: Distribution of the endogenous PRL-1**. Representative immunofluorescence showing the distribution of endogenous PRL-3 in T1 cells treated or not (control) with 10 μg/ml BFA. The fluorescence intensity is shown in pseudocolor with the calibration bar. A zoom of the cells inside white squares is shown in right panels.

**Supplementary Figure 2: Dynamic delivery of GFP-PRL-3 and mCherry-CD3ζ to the IS**. Representative frames of the movie showing a JK cell interacting with a SEE-loaded and CMAC (blue) labelled Raji cell. The green (pseudocolor) and red (grey scale) channels as well as the merged images are shown. The molecule tracked is indicated. Calibration bar of the pseudocolor and the scale bar is indicated. A white arrow and green arrows point to the central and more peripheral areas of the IS, respectively. Numbers indicate time in minutes:seconds.

**Supplementary Figure 3: Distribution of GFP-PRL-3 in non-cognate JK-Raji interactions**. (**A**) A representative cell conjugate of JK cells interacting with CMAC (blue) labelled Raji cells. The green (pseudocolor) and red channels as well as the merged images are shown. Staining for filamentous actin observed in the red channel is indicated. Calibration bar of the pseudocolor is indicated. (**B**) Polarization of GFP-PRL-3 assessed from the increment in fluorescence at the cell-cell interface in comparison with the rest of the cell. Dots indicate individual cells analyzed and the blue line the average value. The dashed line indicates the ratio equals 1 meaning no polarization.

**Supplementary Figure 4. Effect of JMS-053 in the early signaling of primary T lymphoblast**. (**A**) Western blot of protein extracts of primay T lymphoblast stimulated with SEE-loaded Raji cells for the indicated times in minutes (min). Samples of 0 minutes correspond to stimulation times of around the minute required to mix and spin-down cells before protein extraction. SEE- indicates the incubation of lymphoblasts with Raji cells without SEE for the longest time used in stimulated samples. Molecules analyzed are indicated. (**B**) Phosphorylated fraction of the analyzed molecules in (A) normalized to the maximum. The mean +/- the standard deviation is shown (n=3 independent experiments). Samples were compared by a paired two-tailed Student t-test. *p<0.05.

**Movie legend:** **Dynamic delivery of GFP-PRL-3 and mCherry-CD3ζ to the IS**. Representative movie showing a JK cell interacting with a SEE-loaded and CMAC (blue) labelled Raji cell. The green (pseudocolor) and red (grey scale) channels as well as the merged images are shown. Calibration bar of the pseudocolor and the scale bar is indicated. Numbers indicate minutes:seconds.
