# Supplementary figures and images for: Effect of Pharmacological Inhibition of the Catalytic Activity of Phosphatases of Regenerating Liver in Early T Cell Receptor Signaling Dynamics and IL-2 Production
Source: Int J Mol Sci. 2020 Apr 5;21(7):2530. doi: 10.3390/ijms21072530 (PMC7177812; doi:10.3390/ijms21072530)

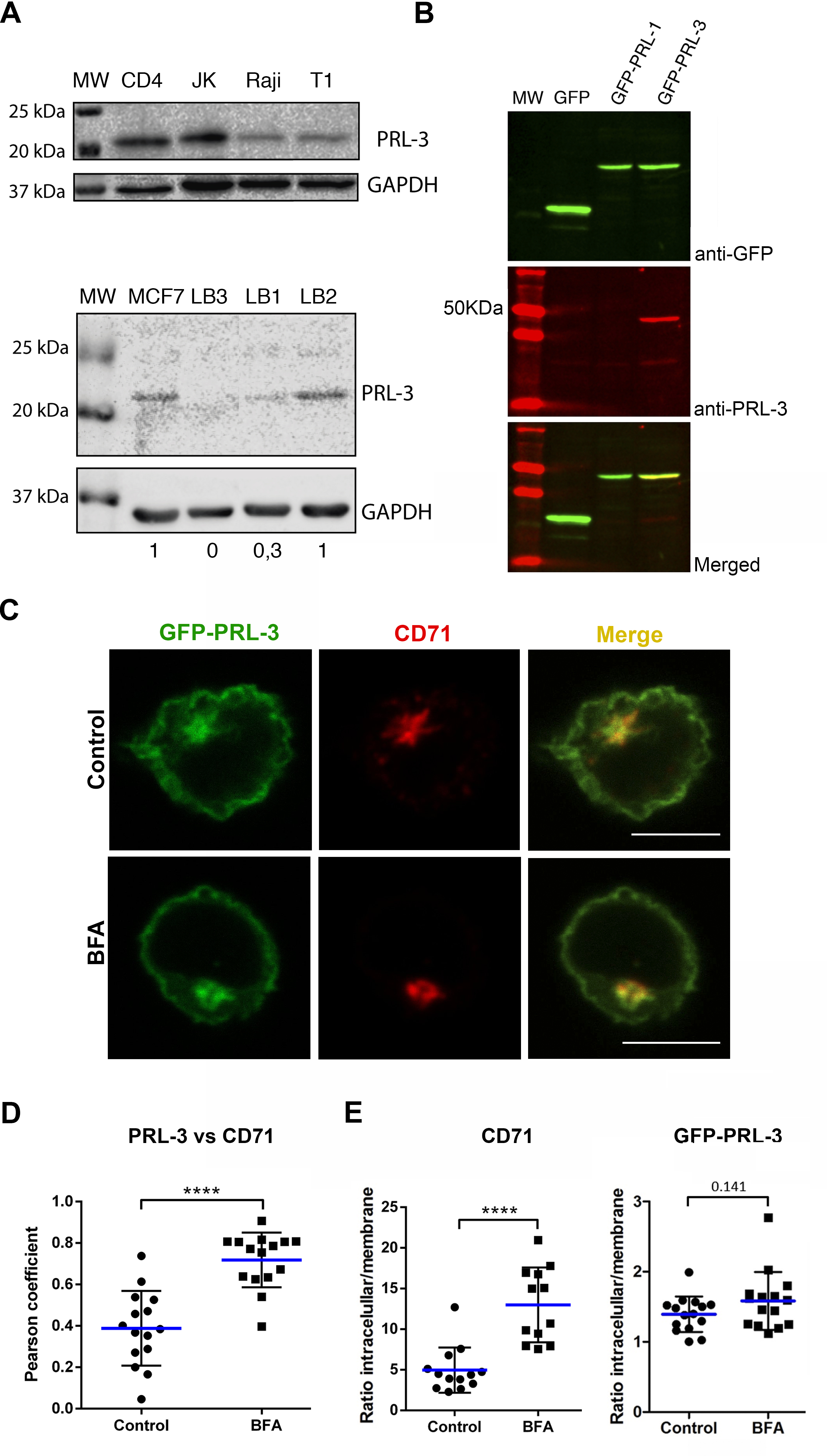

Supplement: Supplementary file 1 [file ijms-21-02530-s001.zip › New_revised_figures/Figure_1_revised.jpg]

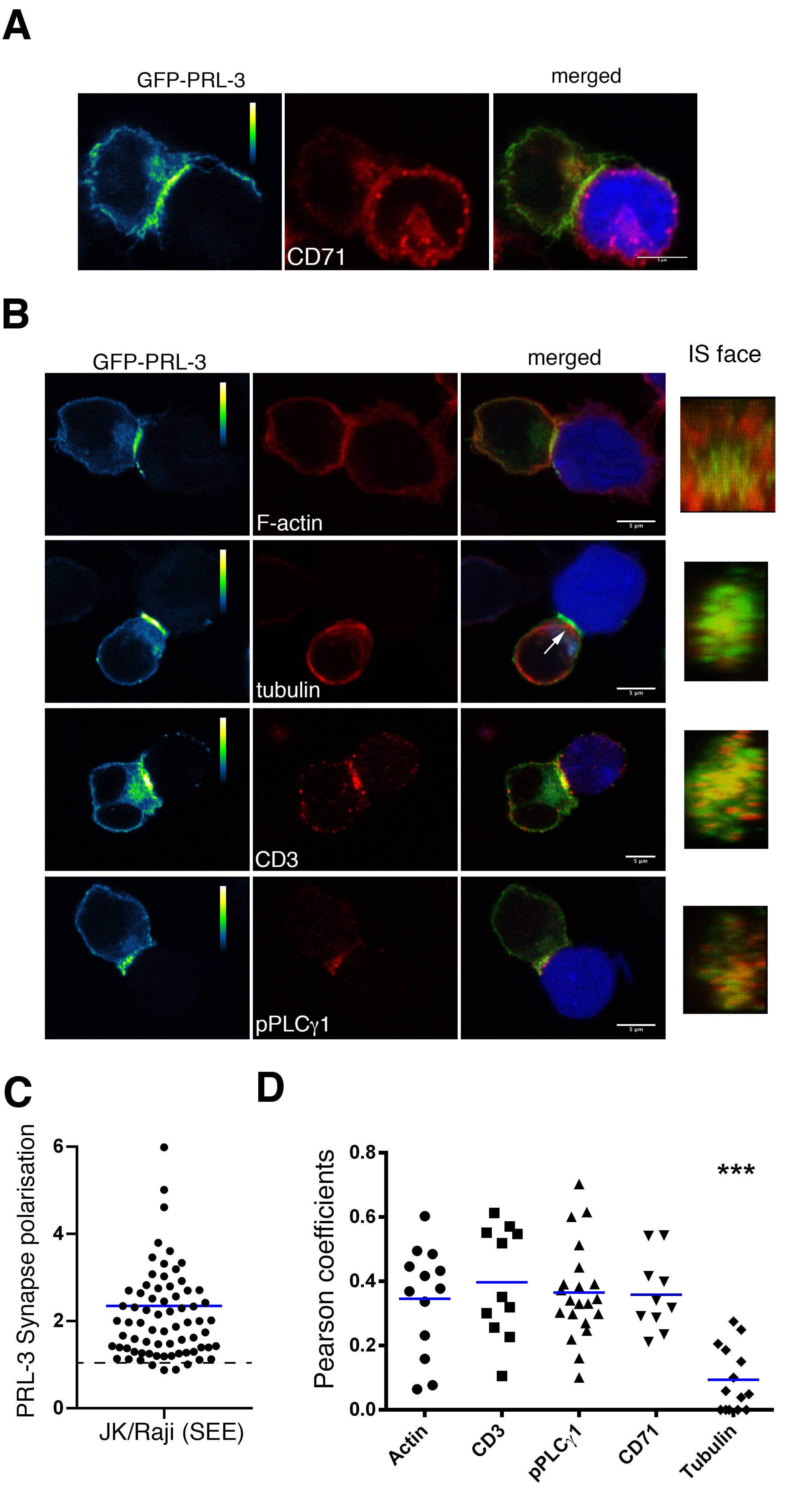

Supplement: Supplementary file 1 [file ijms-21-02530-s001.zip › New_revised_figures/Figure_2_recised.jpg]

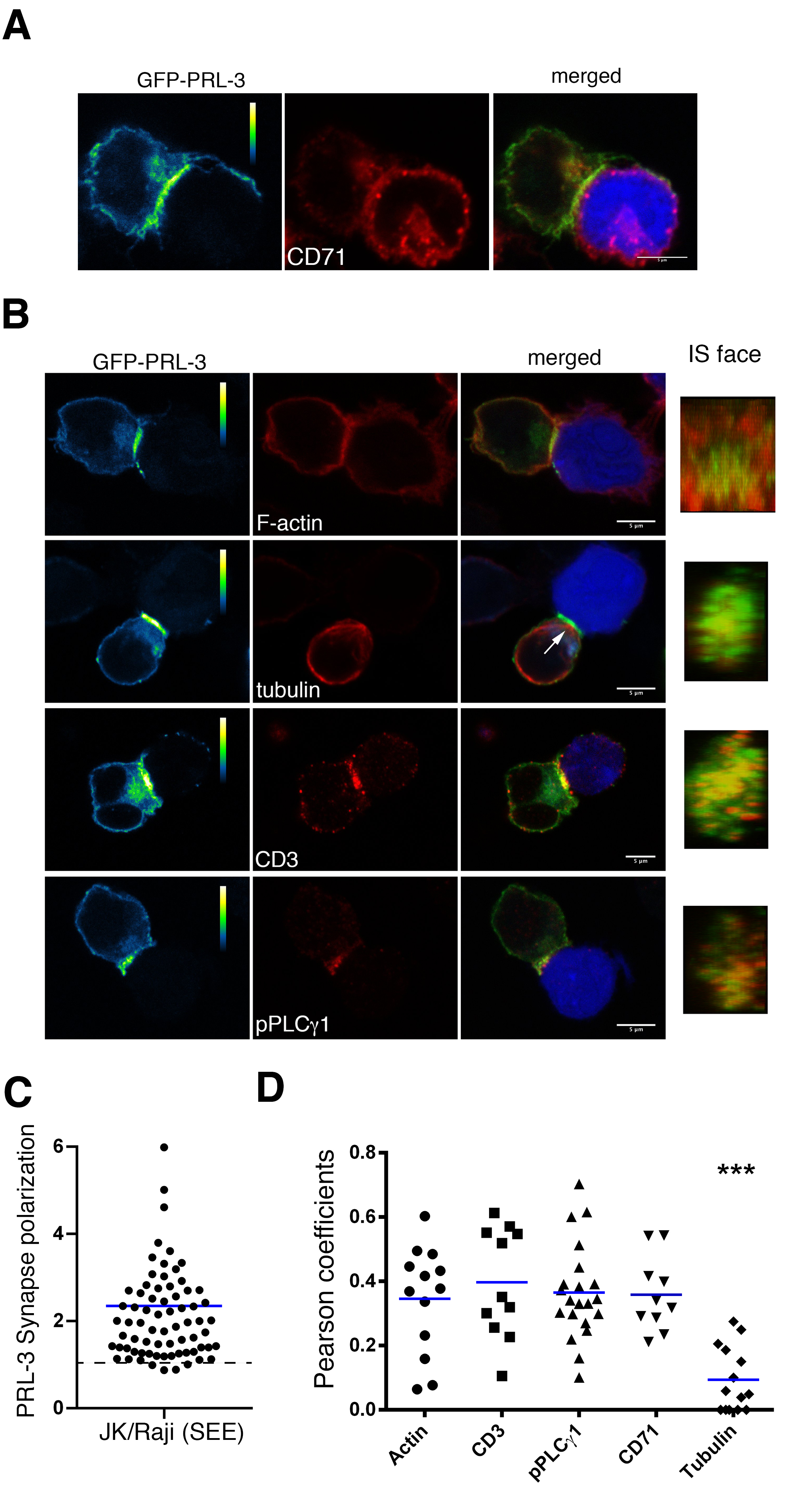

Supplement: Supplementary file 1 [file ijms-21-02530-s001.zip › New_revised_figures/Figure_2_revised.jpg]

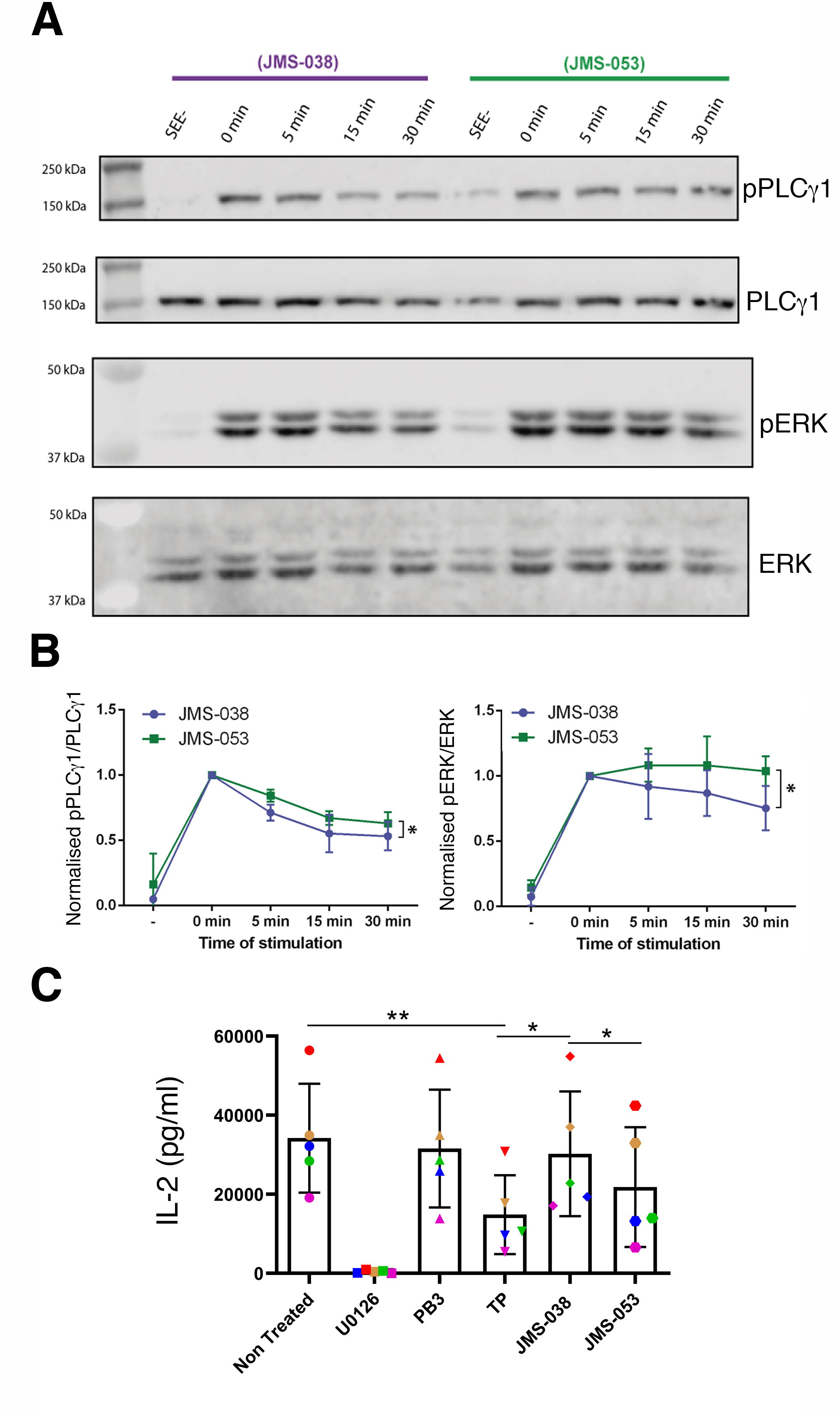

Supplement: Supplementary file 1 [file ijms-21-02530-s001.zip › New_revised_figures/Figure_3_revised.jpg]

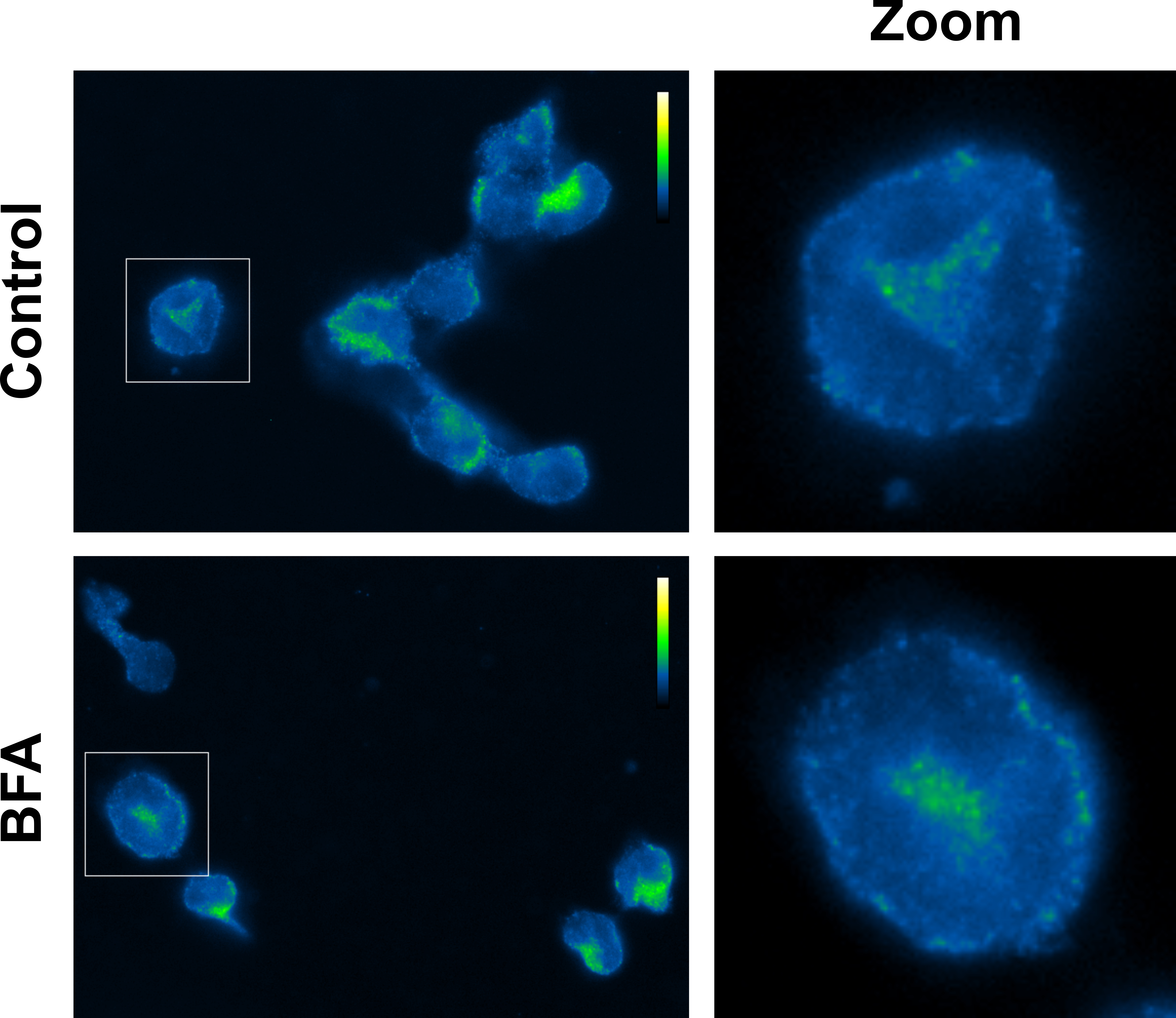

Supplement: Supplementary file 1 [file ijms-21-02530-s001.zip › New_revised_figures/Supplementary_figures/Supplemantary_figure_1.jpg]

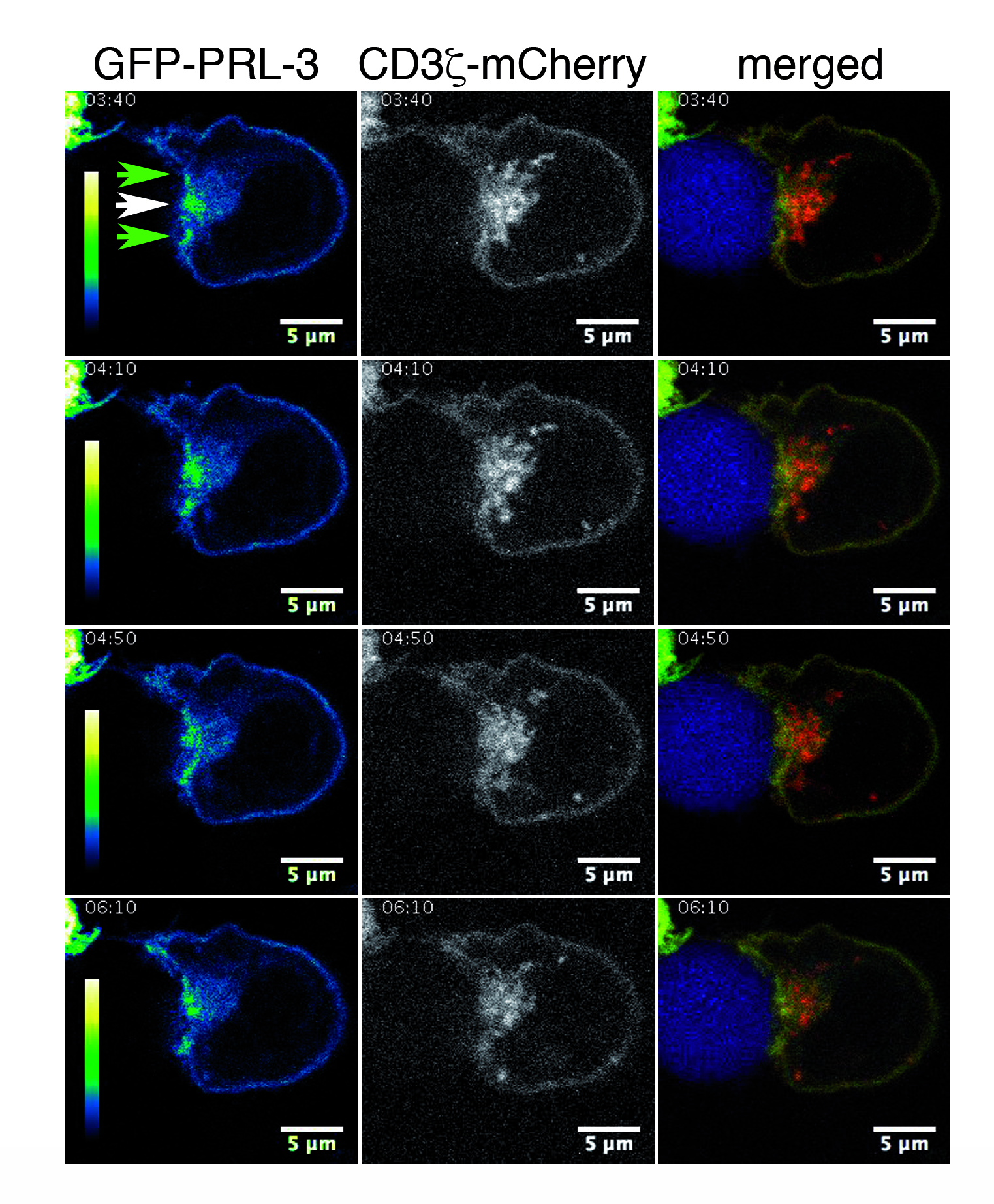

Supplement: Supplementary file 1 [file ijms-21-02530-s001.zip › New_revised_figures/Supplementary_figures/Supplementary_figure_2.jpg]

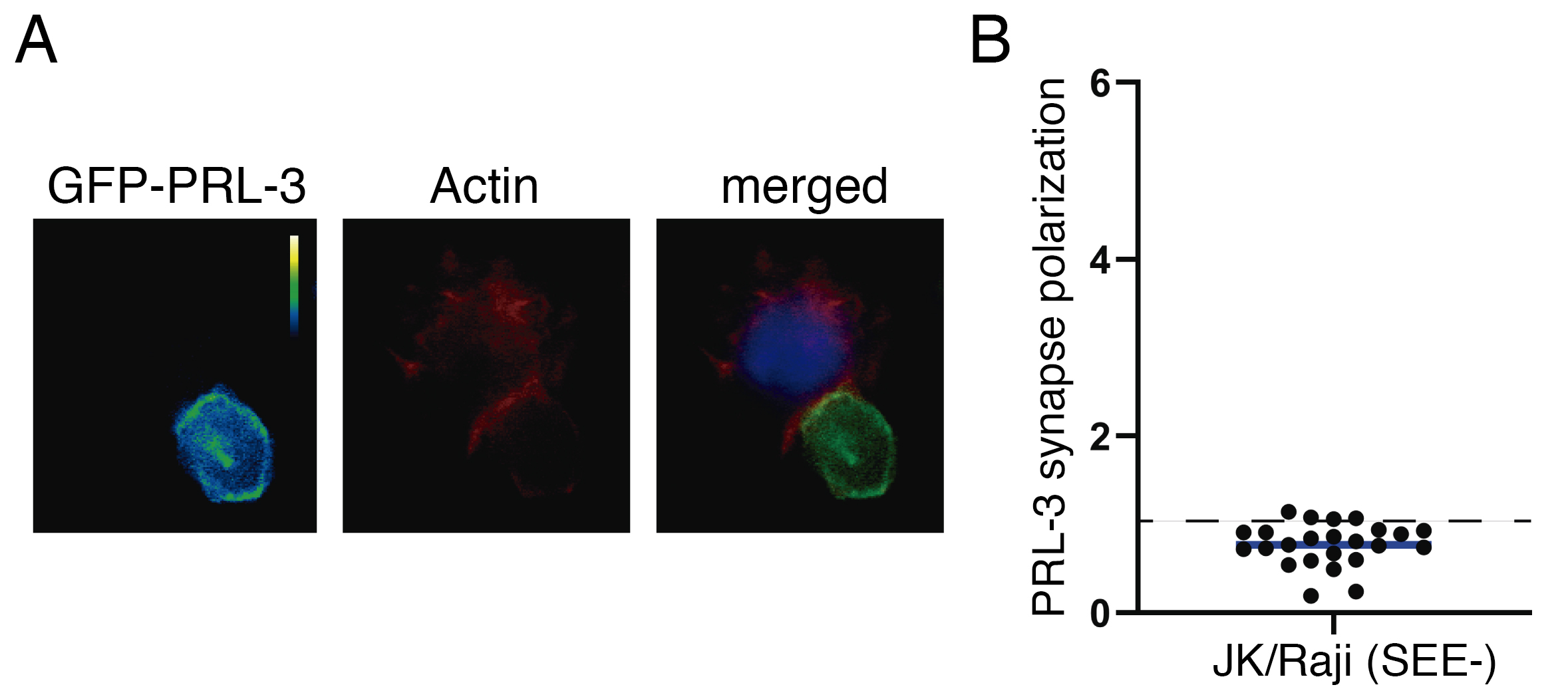

Supplement: Supplementary file 1 [file ijms-21-02530-s001.zip › New_revised_figures/Supplementary_figures/Supplementary_figure_3.jpg]

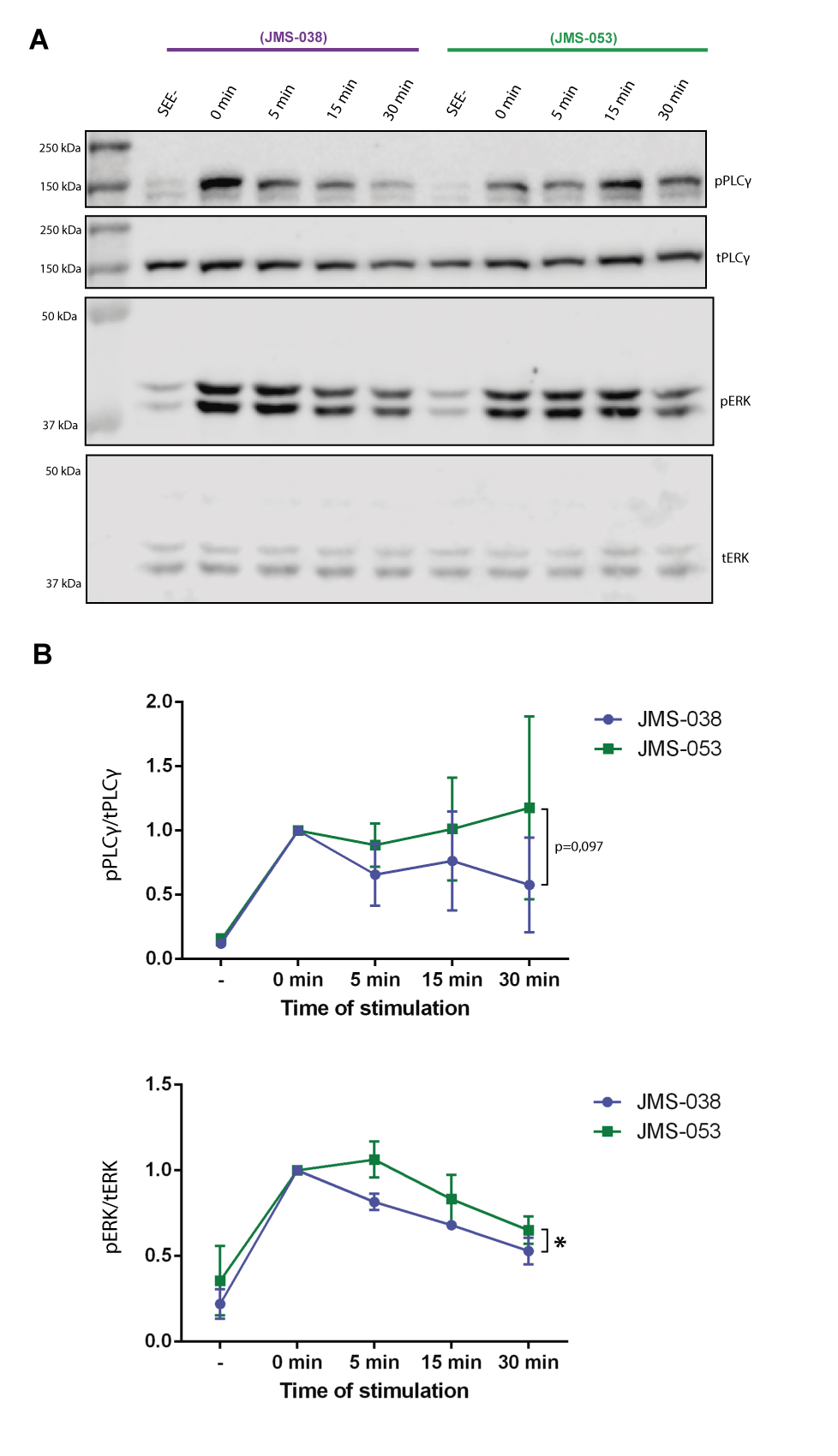

Supplement: Supplementary file 1 [file ijms-21-02530-s001.zip › New_revised_figures/Supplementary_figures/Supplementary_Figure_4.jpg]
